# Geospatial analysis of COVID-19 vaccine access in Kenya: the interplay of travel time, perceived availability, and vaccine uptake

**DOI:** 10.7189/jogh.16.04143

**Published:** 2026-04-10

**Authors:** Jiashuo Sun, Yanjia Cao, Karen Ann Grépin, Tianyu Li, Huanfa Chen, Qunshan Zhao, Primus Che Chi

**Affiliations:** 1 Department of Geography, The University of Hong Kong, Pok Fu Lam, Hong Kong, Hong Kong; 2 Division of Health Economics, Policy and Management, School of Public Health, The University of Hong Kong, Pok Fu Lam, Hong Kong, Hong Kong; 3 Centre for Advanced Spatial Analysis, University College London, London, UK; 4 Urban Big Data Centre, School of Social and Political Sciences, University of Glasgow, Glasgow, UK; 5 Department of Health Systems and Research Ethics, KEMRI-Wellcome Trust Research Programme, Kilifi, Kenya; 6 Directorate of Science and Innovation, Africa Centres for Disease Control and Prevention, Addis Ababa, Ethiopia

## Abstract

**Background:**

COVID-19 vaccines have reduced severe illness and mortality, yet inequitable distribution delayed rollout in many low- and middle-income countries (LMICs), including Kenya, where only 36% of adults were fully vaccinated by October 2022. While sociodemographic and attitudinal determinants of uptake are well documented, role of geographic access remain underexplored at individual level. This study assessed population coverage by travel time to COVID-19 vaccination sites in Kenya and examined how travel time was associated with perceived vaccine availability and vaccine uptake.

**Methods:**

We conducted a secondary analysis of cross-sectional data from Round 3 (October–December 2021) of the COVID-19 Gendered Risks, Impact, and Response Survey. Vaccination site locations were obtained from the Kenyan Ministry of Health. Travel times from constituency population-weighted centroids to the nearest site were estimated using a cost-path distance algorithm in AccessMod. Two binary outcomes were analysed: perceived vaccine availability and vaccine uptake. Logistic regression with restricted cubic splines modelled associations with travel time in the full sample and a restricted sample excluding respondents who reported vaccines as ‘unavailable’ despite modelled travel time <15 minutes.

**Results:**

Nationally, 55% of the population lived within 30 minutes of a vaccination site, 69% within 60 minutes, and 21.6% more than 120 minutes away, with shorter travel times concentrated around major cities and longer times in eastern and northern Kenya. In the full analytic sample, travel time was not significantly associated with perceived availability or uptake. In the restricted sample, longer travel time showed strong, nonlinear associations with lower perceived availability and uptake.

**Conclusions:**

Geographic barriers, captured by modelled travel time, are important determinants of perceived vaccine availability and uptake in Kenya. Combining high-resolution geospatial modelling with individual-level data can guide expansion of vaccination services to improve vaccine equity and preparedness for future health emergencies in Kenya and similar LMICs.

COVID-19 vaccines substantially reduced severe illness and mortality from COVID-19 [1]. Nevertheless, inequities in global distribution impeded timely vaccine access in low- and middle-income countries (LMICs), where mortality risks exceeded those in high-income countries (HICs) by more than 2-fold [2]. By November 2020, HICs – representing just 16% of the global population – had secured 51% of the 7.48 billion doses [3,4]. This disparity delayed LMIC rollouts, resulting in substantially higher vaccination coverage in HICs (80% receiving at least one dose by November 2023) compared to LMICs (33%) [5]. In Kenya, vaccination commenced in March 2021, yet only 36% of adults were fully vaccinated by October 2022 [6,7]. Low vaccination coverage left millions of Kenyans vulnerable to COVID-19 and its comorbidities, exacerbating disruptions to essential health services in the context of the already limited surge capacity of the health care system [8–11]. This was also associated with adverse economic outcomes, including household financial strain and elevated government expenditure for COVID-19 management [12–15].

Multiple individual-level factors influence COVID-19 vaccination behaviours, ranging from demographic and socioeconomic characteristics to information access, trust in government, employment mandates and regular engagement with health care providers [16–24]. Geographic access to vaccination sites, however, remains underexplored as a critical determinant.

Geographic access refers to the ease or difficulty of reaching health care providers from locations where health care services are needed [25]. It significantly affects health care utilisation and health outcomes [26–29]. Methods for assessing access include Euclidean distance [30], two-step floating catchment area approaches [31], and cost-path distance algorithms [32]. The cost-path distance method is particularly effective in sub-Saharan Africa (SSA), as it accounts for real-world conditions such as land cover, terrain, and multiple travel modes (walking, biking, motorised) [33–36]. Empirical evidence links improved geographic access to higher vaccination rates and lower COVID-19 mortality in diverse settings [37–40]. In Kenya, county-level studies have already shown associations between average travel time and vaccine coverage [7].

Nevertheless, most prior research relied on regional or community data, overlooking individual-level heterogeneity in both geographic access and vaccine-related perceptions and behaviours. There is also limited empirical evidence on the direct relationship between travel time and individuals’ perceived vaccine availability, specifically whether longer travel times reduce the likelihood that people perceive vaccines as available. In SSA, while some studies have used survey questions to assess, for example, the percentage of people deterred by long distances to the nearest vaccination point [41] or the amount of time they are willing to travel to receive the vaccine [42], few have assessed the association between travel time and vaccine uptake at the individual level.

To address these gaps, this study was guided by two primary research questions: (1) What proportion of Kenya’s population lived within reach of COVID-19 vaccination sites by travel time thresholds? (2) How was travel time to the nearest vaccination site associated with perceived vaccine availability and vaccine uptake at the individual level?

During the pandemic, as vaccines became more widely available, a significant number of individuals continued to exhibit hesitancy toward vaccination [43,44]. This study aimed to provide critical insights into this phenomenon from the standpoint of geographic access. Using Kenya as a case study, we applied a cost-path distance algorithm to estimate travel times and used restricted cubic splines (RCS) to model their potentially nonlinear association with perceived vaccine availability and vaccine uptake, based on cross-sectional survey data collected between October and December 2021. This analysis provides individual-level evidence from an LMIC that contributes to the global health literature on travel time to vaccination sites, perceived vaccine availability, and COVID-19 vaccination uptake, and may inform public health strategies and equitable resource allocation for future health emergencies in LMICs.

## METHODS

### Study design and data sources

This secondary analysis used de-identified, publicly available cross-sectional data from Round 3 (October–December 2021) of the COVID-19 Gendered Risks, Impact, and Response Survey, which sampled respondents from 258 of Kenya’s 290 constituencies [45]. Locations of COVID-19 vaccination sites (as of August 2021) were obtained from the Kenyan Ministry of Health. Population counts were derived from WorldPop, and geospatial layers (road networks, land cover, digital elevation model (DEM), and water bodies) from established sources. Detailed data descriptions are provided in Appendix S1 in the **Online Supplementary Document**.

**Figure 1:**
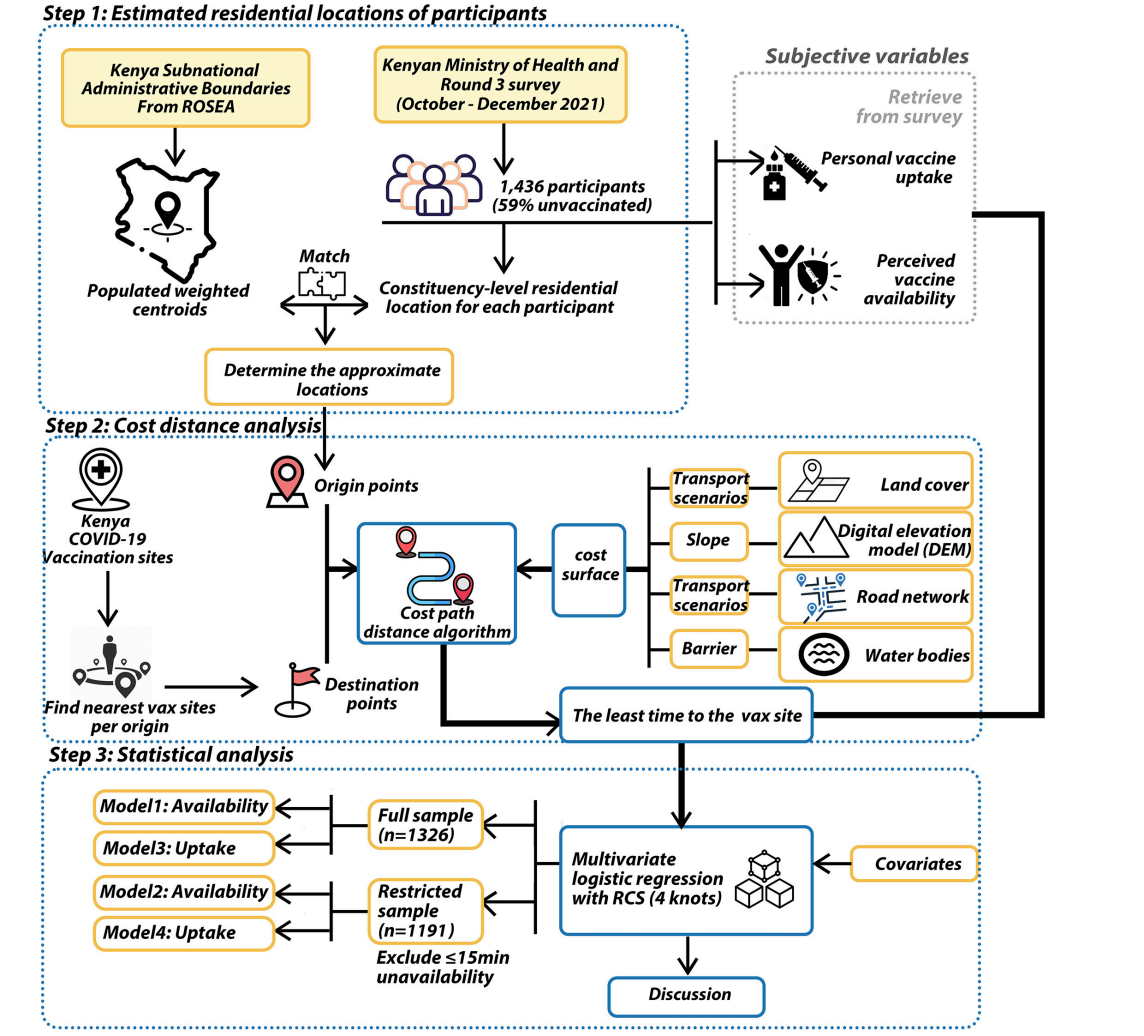
Research framework.

Following the framework in **Figure 1**, analyses proceeded in three steps:

(1) approximating participants’ residential locations by linking them to the population weighted centroid of their constituency;

(2) estimating travel time to the nearest vaccination site using a cost path distance algorithm; and

(3) modelling associations between travel time and perceived vaccine availability and uptake using multivariable logistic regression with travel time modelled by RCS with four knots.

Survey sampling weights and cluster robust standard errors were applied to account for the complex survey design and spatial clustering.

### Calculating travel times

We used the cost-path distance algorithm in AccessMod, version 5.8.3 (World Health Organization) to generate a 100-m resolution cost-friction surface by integrating road networks, land cover, and water body data. Slope data from DEM refined walking and bicycling speeds, while water bodies were treated as barriers unless bridged. The resulting travel time surface represents the shortest theoretical travel time from each grid cell to the nearest vaccination site, assuming optimal conditions and no waiting times, and was then utilised to estimate theoretical minimum travel times from population-weighted centroids of each constituency (Appendix S2 in the **Online Supplementary Document**).

### Outcome variables

We examined two binary outcomes:

1) Perceived COVID 19 vaccine availability: ‘Have COVID-19 vaccines ever been made available to you?’ (1 = available, 0 = unavailable);

2) Vaccine uptake: ‘Have you received at least one dose of a COVID 19 vaccine?’ (1 = yes, 0 = no).

### Predictors and covariates

The predictor was travel time (in minutes) to the nearest vaccination site, calculated from the constituency population weighted centroid and assigned to all respondents in that constituency.

Covariates were selected based on prior literature on COVID 19 vaccination determinants and included: known COVID 19 cases in social networks (proxy for perceived infection risk), number of children in the household, job loss experience, internet connectivity, household wealth index, urban residence, trust in government COVID 19 management, health insurance coverage, educational attainment, age, and gender. Educational attainment was recoded into primary or below, secondary, and post-secondary levels. Urban residence was defined as whether the respondent reported living in an urban location. Known cases and number of children were grouped as none, 1–2, and ≥3. The household wealth index was constructed using principal components analysis of ownership of selected assets, including television, refrigerator, electric iron, fan/electric fan, electricity, and smartphone [46]. Justifications for covariate inclusion are provided in Appendix S3 in the **Online Supplementary Document**.

### Analyses

In primary analyses, multivariate logistic regression was used to examine associations between travel time to the nearest vaccination site and the two binary outcomes: perceived vaccine availability and vaccine uptake. Travel time was modelled using RCS with four knots to capture potential nonlinearity, informed by prior literature on geographic access and health care utilisation [47–49].

To address potential ambiguity in the self-reported measure of perceived vaccine availability, which may reflect multiple constructs including eligibility, awareness, local supply, or other barriers beyond geographic proximity, models were fitted in both the full analytic sample and a restricted sample. The restricted sample excluded respondents who reported vaccine unavailability despite modelled travel time less than 15 minutes. We specified four models: Model 1 (full sample, perceived vaccine availability), Model 2 (restricted sample, perceived vaccine availability), Model 3 (full sample, vaccine uptake), and Model 4 (restricted sample, vaccine uptake).

As spline coefficients are not directly interpretable, predicted probabilities from the RCS models are plotted to display the shape of the associations. We used design-based Wald tests to assess the overall association of travel time with each outcome, and design-based Rao-Scott likelihood ratio tests to assess deviation from linearity (the nonlinearity component). Multicollinearity was assessed using variance inflation factors (VIF). All models adjusted for covariates listed in the Predictors and Covariates section. 

We assessed the spatial autocorrelation after adjustment for travel time and other covariates by computing survey weighted constituency level Pearson residuals from the models for both vaccination status and vaccine availability. Residuals were averaged within constituencies using the survey weights, and Moran’s I was calculated using a contiguity based spatial weights matrix derived from Kenyan constituency boundaries.

Three sensitivity analyses were conducted to assess the robustness of the findings. First, we compared respondents excluded from the restricted sample with those retained, examining all covariates from the main regression models. Continuous variables were summarised as means and standard deviations and compared using Wilcoxon rank sum tests; categorical variables were summarised as proportions and compared using Pearson χ^2^ tests.

Second, to account for clustering within constituencies and potential unmeasured constituency level heterogeneity, we refitted all primary models using mixed effects logistic regression with a random intercept for constituency, retaining the same fixed effects and covariates as in the main analyses.

Third, to assess sensitivity to the definition of travel time, we redefined the exposure using grid-based measures. Using the travel time surface, we calculated, for each constituency, the mean and median of the shortest travel time from all grid cells; these mean and median values were then assigned to respondents and substituted for the centroid based measure in otherwise identical models. All analyses were conducted in *R*, version 4.2.2 (R Foundation for Statistical Computing, Vienna, Austria).

## RESULTS

### Travel time surface and population coverage

**Figure 2:**
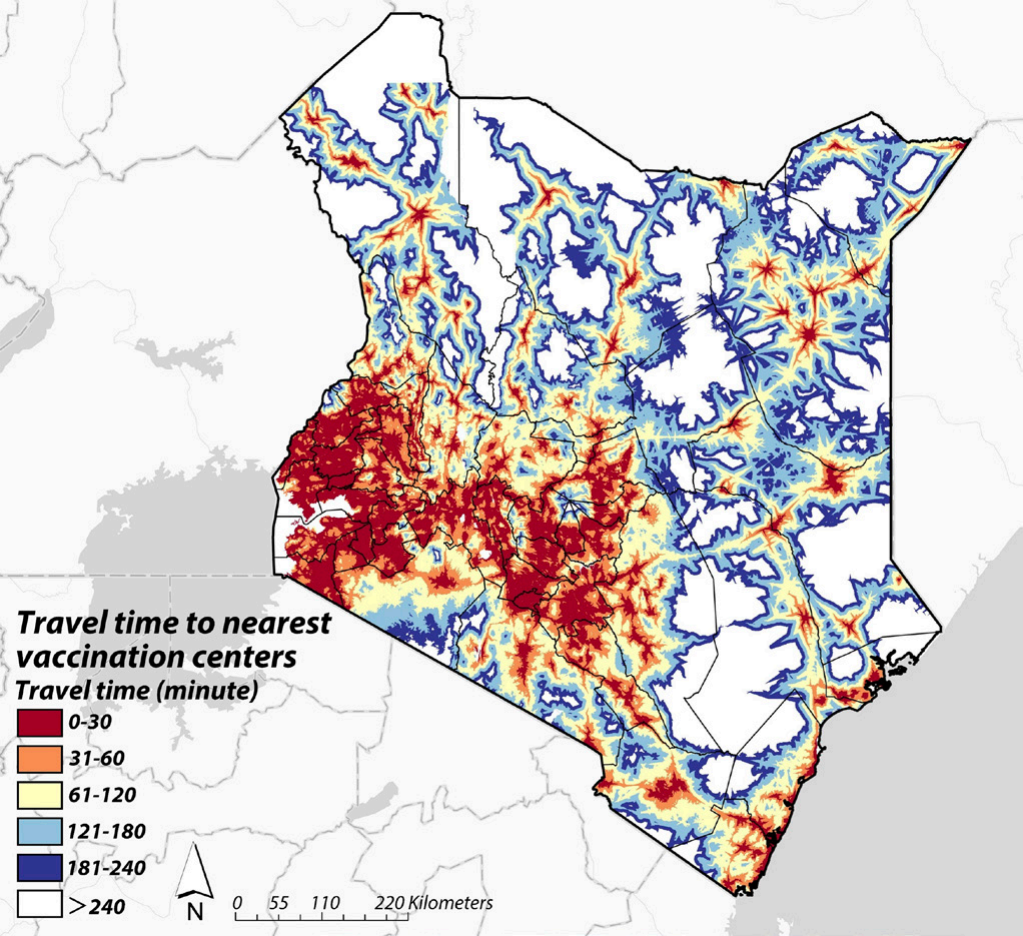
Shortest travel time to the nearest vaccination centres.

The cost-path distance algorithm produced a surface illustrating the shortest theoretical travel times to the nearest vaccination sites for every populated area in Kenya (**Figure 2**). Regional patterns showed shorter travel times near Nairobi and the major cities of Eldoret, Kisumu, and Mombasa. In contrast, eastern and northern regions generally showed longer travel times. Most individuals in these regions, except those near towns, showed travel times exceeding two hours to the nearest vaccination site.

Regarding population coverage, about 30.4 million people (55% of the total population) lived within 30 minutes of a vaccination site. Around 38.2 million (69%) lived within 60 minutes, while about 12 million (21.6%) lived more than 120 minutes away.

### Associations between travel time and vaccine outcomes

Among the 1436 survey respondents, 846 (59%) had not received any COVID-19 vaccination at the time of the survey. The percentage distribution of vaccinated respondents is illustrated in Figure S3 in the **Online Supplementary Document**. After excluding 110 respondents with missing data on educational attainment, the full analytic sample comprised 1326 individuals. In the restricted sample, an additional 135 respondents were excluded due to reporting vaccine unavailability despite modelled travel time less than 15 minutes, resulting in 1191 individuals for Models 2 and 4.

Travel time to the nearest vaccination site was not associated with perceived vaccine availability in the full analytic sample but showed a strong and nonlinear association in the restricted sample (**Table 1**). In the full sample for perceived vaccine availability (Model 1), the design-based Wald test for the overall association of travel time was not statistically significant (*P* = 0.34), and the design-based Rao-Scott likelihood ratio test provided no evidence of departure from linearity (*P* = 0.47). In the restricted sample (Model 2), both the overall association (*P* < 0.001) and the nonlinearity test (*P* < 0.001) were highly statistically significant.

**Table 1:** Overall and nonlinear association tests for travel time using RCS (4 knots)

Model	Overall association (design-based Wald F (df_1_, df_2_), *P*-value)	Nonlinearity Test (Rao-Scott LRT 2logLR, df, *P-*value)
Model 1 perceived vaccine availability (full analytic sample)	F (3, 239) = 1.12, *P* = 0.34	2logLR = 1.50, df = 2, *P* = 0.47
Model 2 perceived vaccine availability (restricted sample)	F (3, 230) = 24.00, *P* < 0.001	2logLR = 82.31, df = 2, *P* < 0.001
Model 3 vaccine uptake (full analytic sample)	F (3, 239) = 1.05, *P* = 0.37	2logLR = 1.22, df = 2, *P* = 0.53
Model 4 vaccine uptake (restricted sample)	F (3, 230) = 3.71, *P* = 0.012	2logLR = 6.58, df = 2, *P* = 0.039

design-based Wald F – design-based Wald F statistic, df – degrees of freedom, LRT – Likelihood Ratio Test, RCS – restricted cubic splines, 2logLR – twice the log-likelihood ratio

For vaccine uptake, travel time to the nearest vaccination site was not associated with uptake in the full analytic sample, but showed a statistically significant and nonlinear association in the restricted sample. In the full sample (Model 3), the design-based Wald test for the overall association of travel time with vaccine uptake was not statistically significant (*P* = 0.37), and the Rao-Scott likelihood ratio test provided no evidence of nonlinearity (*P* = 0.53). In the restricted sample (Model 4), both the overall association (*P* = 0.012) and the nonlinearity test (*P* = 0.039) were statistically significant. All independent variables showed VIF values below 5 in all four models, indicating low multicollinearity.

**Figure 3:**
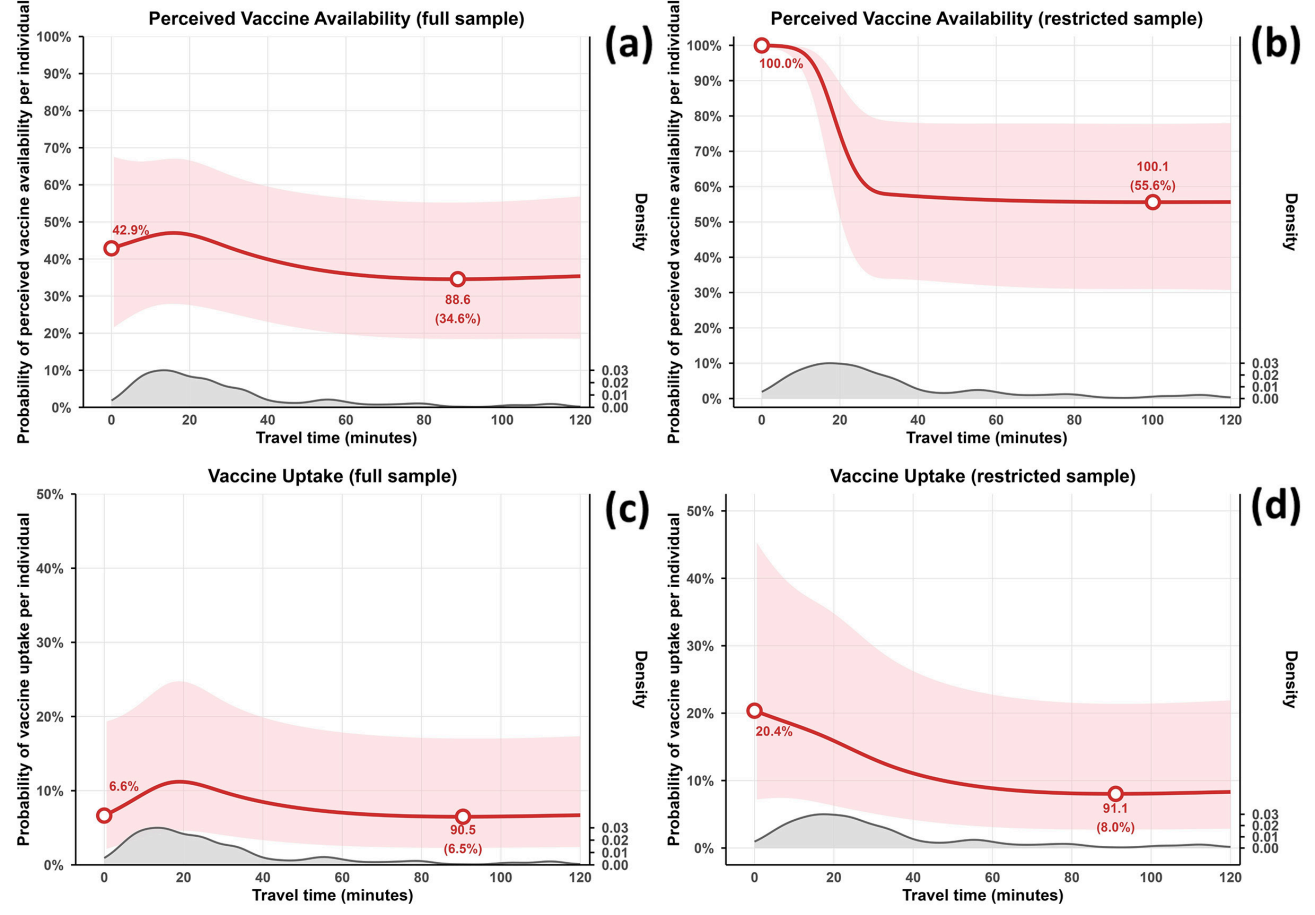
Predicted probabilities from RCS as functions of travel time to the nearest COVID 19 vaccine site. **Panel A**. Probability of perceived vaccine availability in the full sample (Model 1). **Panel B**. Probability of perceived vaccine availability in the restricted sample (Model 2). **Panel C**. Probability of vaccine uptake in the full sample (Model 3). **Panel D**. Probability of vaccine uptake in the restricted sample (Model 4).

Predicted probabilities from the RCS models are presented in **Figure 3**. Density plots at the bottom of each panel illustrate the empirical distribution of individual travel times, underscoring the relative sparsity of observations at the very long durations. In the full sample (Model 1) (**Figure 3**, Panel A), the predicted probability of perceived vaccine availability started at approximately 42.9% for very short travel times, rose with increasing time over roughly the first 18 minutes, then declined to a minimum of about 34.6% at approximately 88.6 minutes, and remained relatively stable at longer travel times. For the restricted sample (Model 2) (**Figure 3**, Panel B), the predicted probability of perceived vaccine availability approached 100% at minimal travel times, dropped sharply across the initial 30 minutes to around 58%, and subsequently tapered off more slowly to a low of about 55.6% at roughly 100.1 minutes before levelling out. Regarding vaccine uptake in the full sample (Model 3) (**Figure 3**, Panel C), the predicted probability began at approximately 6.6% for very short travel times, increased up to about 20 minutes, then fell to a minimum of around 6.5% at 90.5 minutes, and stayed relatively constant. In the restricted sample (Model 4) (**Figure 3**, Panel D), the predicted probability commenced at about 20.4% for minimal travel times, decreased to approximately 8.0% at around 91.1 minutes, and then plateaued at longer travel times. Adjusted odds ratios (aOR) and 95% confidence intervals for covariates in the multivariable models are presented in **Table 2**. Higher age consistently showed significant positive associations across all models. Greater perceived infection risk and strong trust in government were positively associated in most models, while insurance coverage exhibited significant positive effects in the vaccine uptake models.

**Table 2:** Adjusted aORs and 95% CIs for covariates associated with perceived vaccine availability and vaccine uptake

	Model 1 perceived vaccine availability		Model 2 perceived vaccine availability		Model 3 vaccine uptake		Model 4 vaccine uptake	
**Variable**	**AOR (95% CI)**	***P*-value**	**AOR (95% CI)**	***P*-value**	**AOR (95% CI)**	***P*-value**	**AOR (95% CI)**	***P*-value**
Age	1.031 (1.010, 1.054])	0.004	1.041 (1.014, 1.068)	0.003	1.044 (1.023, 1.065)	<0.001	1.047 (1.024, 1.070)	<0.001
Perceived infection risk								
*1–2*	1.241 (0.724, 2.130)	0.431	0.930 (0.506, 1.710)	0.815	1.660 (1.031, 2.675)	0.037	1.451 (0.890, 2.363)	0.135
*3+*	1.694 (1.049, 2.735)	0.031	1.334 (0.769, 2.315)	0.304	1.977 (1.250, 3.127)	0.004	1.609 (1.012, 2.557)	0.044
Job loss experience	0.820 (0.526, 1.278)	0.379	0.779 (0.454, 1.338)	0.364	1.284 (0.845, 1.950)	0.241	1.313 (0.855, 2.016)	0.213
Number of children in the household								
*1–2*	1.493 (0.884, 2.519)	0.133	0.887 (0.469, 1.678)	0.711	1.100 (0.663, 1.826)	0.711	0.830 (0.494, 1.395)	0.48
*3+*	0.933 (0.560, 1.555)	0.79	0.514 (0.278, 0.951)	0.034	0.900 (0.519, 1.562)	0.708	0.706 (0.391, 1.277)	0.249
Internet connectivity	0.756 (0.391, 1.461)	0.404	1.055 (0.458, 2.431)	0.9	1.349 (0.762, 2.391)	0.303	1.604 (0.858, 2.999)	0.138
Trust in government								
*Somewhat distrust*	1.003 (0.458, 2.198)	0.994	0.744 (0.298, 1.857)	0.524	1.018 (0.500, 2.074)	0.96	1.032 (0.497, 2.144)	0.932
*Neither trust nor distrust*	1.115 (0.478, 2.599)	0.801	0.621 (0.213, 1.806)	0.38	0.833 (0.319, 2.177)	0.709	0.710 (0.256, 1.968)	0.508
*Somewhat trust*	1.557 (0.821, 2.953)	0.174	1.064 (0.464, 2.441)	0.884	1.943 (1.053, 3.583)	0.034	1.816 (0.960, 3.438)	0.067
*Strongly trust*	1.926 (1.033, 3.591)	0.039	1.988 (0.938, 4.212)	0.073	3.492 (1.817, 6.712)	<0.001	3.718 (1.846, 7.487)	<0.001
Insurance coverage	1.347 (0.863, 2.103)	0.189	0.934 (0.533, 1.637)	0.811	1.823 (1.232, 2.699)	0.003	1.816 (1.208, 2.730)	0.004
Urban residence	0.919 (0.578, 1.459)	0.718	1.172 (0.658, 2.086)	0.588	0.892 (0.589, 1.351)	0.588	0.966 (0.637, 1.465)	0.871
Wealth	1.084 (0.882, 1.332)	0.44	1.070 (0.819, 1.398)	0.618	0.958 (0.750, 1.223)	0.728	0.919 (0.704, 1.198)	0.53
Educational attainment								
*Secondary*	1.252 (0.668, 2.346)	0.482	1.425 (0.618, 3.287)	0.404	1.709 (0.904, 3.231)	0.099	1.719 (0.922, 3.204)	0.088
*Post-secondary*	1.566 (0.861, 2.849)	0.141	1.471 (0.679, 3.187)	0.326	1.639 (0.861, 3.120)	0.132	1.560 (0.808, 3.013)	0.185
Male	1.115 (0.729, 1.706)	0.614	1.041 (0.620, 1.746)	0.88	0.948 (0.640, 1.405)	0.791	0.899 (0.598, 1.352)	0.607

AOR – adjusted odds ratios, CI – confidence interval

Moran’s I statistics computed from the survey weighted constituency level Pearson residuals provided no evidence of residual spatial autocorrelation after adjusting for travel time and other covariates in any of the four models (**Table 3**). These findings indicate that unmodelled spatial structure is unlikely to bias our estimates and support the robustness of the fitted survey weighted logistic regression models.

**Table 3:** Moran's I statistics for residual spatial autocorrelation in primary analysis

Model	Sample	Outcome	Moran's I	*P*-value
Model 1	Full	Perceived Vaccine Availability	−0.005	0.509
Model 2	Restricted	Perceived Vaccine Availability	−0.001	0.47
Model 3	Full	Vaccine Uptake	−0.009	0.545
Model 4	Restricted	Vaccine Uptake	−0.006	0.524

### Sensitivity analyses

Across all sensitivity analyses, the main findings were broadly robust. Respondents excluded from the restricted analytic sample were younger, less educated, poorer, less likely to have health insurance, and more likely to report no known COVID 19 cases in their social networks than those retained, indicating that the exclusion criteria removed a systematically more disadvantaged subgroup whose responses on ‘availability’ likely reflected barriers beyond geographic access. Mixed effects logistic regression models with constituency level random intercepts produced a similar pattern to the primary survey weighted models: in the full sample, travel time was not associated with perceived vaccine availability or uptake, whereas in the restricted sample longer travel time remained strongly and nonlinearly associated with both outcomes. When mean or median grid-based travel times were used in place of population weighted centroids, travel time showed no clear association with vaccine uptake in any specification, but the strong nonlinear association with lower perceived availability in the restricted sample persisted (Appendix S4 in the **Online Supplementary Document**).

## DISCUSSION

This study estimated theoretical minimum travel times from constituency population weighted centroids to the nearest COVID 19 vaccination site in Kenya using a cost path distance algorithm, and examined how these travel times were associated with perceived vaccine availability and vaccine uptake. Travel time patterns were highly heterogeneous: shorter times clustered around Nairobi and other major cities, whereas much of eastern and northern Kenya exhibited substantially longer travel times. The result was consistent with prior evidence that COVID-19 vaccination sites were primarily located in high-population-density areas of Kenya [7]. Residents in these areas typically could access multiple COVID-19 vaccination sites. More than half of the population lived within 30 minutes of a vaccination site, while about one fifth were more than two hours away. These findings reinforce the unequal spatial distribution of vaccination services and suggest that strategies that bring services closer to underserved communities, such as targeted expansion of fixed sites or deployment of mobile and outreach services, may merit consideration as part of broader efforts to improve coverage. Our results provide an empirical basis for subsequent evaluations of the feasibility, costs, workforce requirements, and comparative performance of such delivery models.

In individual level analyses using population weighted centroids, travel time was not associated with perceived vaccine availability or uptake in the full analytic sample, but showed clear nonlinear associations in the restricted sample that excluded respondents reporting vaccine ‘unavailability’ despite a modelled travel time under 15 minutes. In this restricted sample, the predicted probability of perceiving vaccines as available was very high at short travel times and declined steeply over the first 30 minutes before plateauing. Vaccine uptake also declined with increasing travel time up to roughly 90 minutes, with relatively little change at longer durations. These nonlinear associations are consistent with previous work on geographic access and health care service utilisation [47–49]. One plausible interpretation is that as the time required to reach a vaccination site increases, geographic access becomes a more salient barrier for a subset of individuals [41], which is reflected in both lower perceived availability and lower uptake.

The comparison of excluded and retained respondents helps to clarify why associations differed between the full and restricted samples, and why the restricted sample is better suited for examining geographic access. Respondents excluded from the restricted sample were, on average, younger, less educated, from poorer households, more often uninsured, and reported lower perceived infection risks than those retained. For this subgroup, the survey item on vaccine ‘availability’ plausibly reflects constructs beyond geographic proximity, including perceived eligibility, information or communication gaps, and barriers related to engagement with the formal health system. Retaining these respondents in the full sample therefore introduces substantial heterogeneity in how ‘availability’ is interpreted, diluting the extent to which the measure reflects geographic access. By contrast, the restricted sample is likely to provide a clearer signal of the relationship between modelled travel time and both perceived availability and uptake, which is consistent with the stronger and statistically significant associations observed in the restricted sample models.

Sensitivity analyses generally supported the robustness of the main findings. Mixed effects logistic regression models with constituency level random intercepts yielded a similar pattern: in the restricted sample, travel time remained strongly and nonlinearly associated with both perceived availability and uptake, whereas associations in the full sample remained weak and non-significant. This suggests that the primary results are not driven by unmeasured constituency level characteristics captured by a random intercept.

When we re estimated the survey weighted models using mean or median grid-based travel times instead of population weighted centroids, the results were only partly consistent with the primary findings. In the restricted sample, longer grid-based travel times remained strongly and nonlinearly associated with lower perceived availability, supporting the robustness of this association. In contrast, grid-based measures showed no clear association with vaccine uptake. One plausible explanation is that mean and median travel times summarise conditions across all grid cells within the relevant constituency, including unpopulated areas such as national parks and nature reserves. This is particularly problematic in large, geographically heterogeneous constituencies in northern and eastern Kenya, where extensive uninhabited or sparsely populated areas can inflate or increase the variability of travel times in ways that do not reflect most residents’ actual access. Population weighted centroids, by comparison, give greater weight to locations where people reside and are less influenced by extreme travel times in uninhabited areas, making them a more appropriate and precise proxy for the typical location of survey respondents within constituencies [50].

This study contributes to the literature by integrating cost path distance modelling with individual level survey data in an LMIC setting. To our knowledge, it is among the first individual level analyses to relate modelled travel time to perceived vaccine availability. By jointly examining travel time, perceived availability, vaccine uptake, and individual and household characteristics, the study moves beyond purely area level analyses and provides new evidence on how physical access barriers are associated with vaccination related perceptions and behaviours. The use of RCS enabled us to capture nonlinear associations between travel time and both outcomes, which may be missed by linear specifications. Together, these features offer a framework for future work on how geographic access interacts with individual level determinants of vaccination in Kenya and similar LMIC contexts.

Several limitations should be considered. First, because precise residential addresses were unavailable, respondents’ locations and travel times could only be approximated at the constituency level. Although population weighted centroids are likely to be a reasonable proxy for the location of most individuals in a constituency, misclassification of travel time exposure is still possible. Sensitivity analyses indicated that associations with vaccine uptake were more sensitive to the choice of travel time metric than associations with perceived availability, suggesting that more spatially precise residence data could strengthen future analyses. Second, the cost path distance algorithm estimates theoretical minimum travel times under ideal conditions and does not account for monetary costs of transport, seasonal road degradation, congestion, insecurity, or waiting times [22,36,51,52]. Actual travel times may therefore be longer than those modelled here. Third, vaccination site locations were obtained from the Ministry of Health registry as of August 2021, which already included many mobile and outreach sites, but additional changes before or during the October–December survey period may have altered the service environment in ways we could not capture. In areas where new sites were added after August, our model likely overestimated travel times and underestimated population coverage. Fourth, the analysis is based on cross-sectional survey data, with trust in government and vaccination outcomes measured at the same time. This design precludes establishing temporal ordering and raises the possibility of endogeneity. Although we adjusted for a range of observed covariates, some potentially important determinants, such as opportunities for vaccination through workplaces, ethnicity, informal settlement residence, and local health system capacity, were not available. Future research should, where feasible, incorporate more granular spatial data on residence, richer contextual variables, and longitudinal designs to better characterise the temporal dynamics and potential causal pathways linking geographic access, perceptions of availability, and vaccine uptake.

## CONCLUSIONS

This study provides evidence that geographic access, operationalised as modelled travel time to the nearest COVID 19 vaccination site, is associated with both perceived vaccine availability and vaccine uptake in Kenya when travel times are calculated from constituency population weighted centroids and analyses exclude respondents who lived within 15 minutes of a vaccination site but reported vaccines as ‘unavailable’. By integrating cost path distance modelling with individual level survey data and applying RCS, we identified nonlinear associations and documented substantial within country inequalities, with residents of eastern and northern regions experiencing markedly longer travel times than those in major cities. These findings underscore the value of combining geospatial analysis with individual level data to identify areas where geographic access to vaccination is most constrained and to guide broad geographic targeting of future vaccination efforts. Decisions about whether and how to expand services will require complementary evidence from economic evaluations, feasibility assessments, and implementation studies.

## Additional material


Online Supplementary Document
