# Supplementary material for: Geospatial analysis of COVID-19 vaccine access in Kenya: the interplay of travel time, perceived availability, and vaccine uptake
Source: J Glob Health. 2026 Apr 10;16:04143. doi: 10.7189/jogh.16.04143 (PMC13067299; doi:10.7189/jogh.16.04143)
Supplement: Online Supplementary Document [file jogh-16-04143-s001.pdf]

**Supplement to: Sun J, Cao Y, Grépin KA, Li T, Chen H, Zhao Q, Chi PC. Geospatial analysis of COVID-19 vaccine access in Kenya: the interplay of travel time, perceived availability, and vaccine uptake. J Glob Health. 2026;16:04143.**

**Contents**

**Appendix S1. Overview of data collected for this study.** .................................................... 2
**Appendix S2. Calculating travel times.** ................................................................................ 3
**Appendix S3. Justification for covariate selection.** ............................................................. 4
**Appendix S4. Results from Sensitivity analyses.** ................................................................. 5
**Appendix S5. Supplementary figures and tables.** ................................................................ 7
Figure S1. Distribution of COVID-19 vaccination sites across Kenya. .................................... 7
Figure S2. Hypothesized modes of transportation and corresponding speeds for residents traveling across different land covers or road types and land cover distribution across Kenya. ........................................................................................................................................... 8
Figure S3**.** Percentage of survey respondents who have been vaccinated across Kenya. ........ 9
Table S1. Characteristics of Respondents Excluded From and Retained in the Restricted Analytic Sample. ......................................................................................................... 10
Table S2. Associations Between Travel Time, Perceived Vaccine Availability, and Vaccine Uptake in Mixed-Effects Logistic Regression Models. ........................................................ 11
Table S3. Associations Between Travel Time and Vaccine Outcomes Using Grid-Based Mean and Median Travel Time Measures. ...................................................................... 12

**Appendix S1. Overview of data collected for this study.**

Vaccination site data were obtained from the Kenyan Ministry of Health (August 2021), the most recent publicly available list for 2021. Site addresses (e.g., Chwele Sub-County Hospital, Kabuchai, Bungoma County) were geocoded using Google Maps Application Programming Interface, based on the WGS-84 coordinate system [1]. An examination of earlier publicly available list from March to June 2021 showed that the vaccination site registry expanded significantly by August. The earlier list included 622 sites and were restricted to traditional healthcare facilities such as dispensaries, health centres, and hospitals. In contrast, the August 2021 list used in this study increased by 189 sites and incorporated additional mobile or outreach vaccination activities (e.g., Anti Stock Theft Unit in Gilgil, Nakuru; Ruiru GK Prison in Ruiru, Kiambu). Therefore, as shown in Supplementary Figure S1, the location of 811 vaccination sites in Kenya were collected and used to calculate travel times.

To protect the privacy of survey participants, their residential locations were documented at the constituency level, and the population-weighted centroid of each constituency was used as an approximate representation of the respondents' origins. No personal or identifiable information was used, and data usage complied with the data-sharing policies of the respective organizations.

Population data were collected from WorldPop [2] for Kenya in 2020 to evaluate the population coverage of vaccination centers and compute the population-weighted centroids. This dataset provides estimates of the total population per grid cell at a spatial resolution of 100 meters. Kenya subnational boundaries were collected from Regional Office for Southern and Eastern Africa (ROSEA) of United Nations Office for the Coordination of Humanitarian Affairs [3]. Additionally, we collected four types of geospatial data to quantify travel impedance between the estimated residential locations and corresponding nearest vaccination sites. Road network data from the Kenya Roads Board included primary, secondary, county, and rural roads [4–6]. Land cover data for 2021 were obtained from the European Space Agency at 10-meter resolution [7]. This dataset includes eight land cover classifications: water, trees, flooded vegetation, crops, built-up areas, bare ground, snow/ice, and rangeland. Digital Elevation Model (DEM) data at 30-meter spatial resolution were obtained from the Shuttle Radar Topographic Mission (SRTM) via the Regional Center for Mapping of Resources for Development (RCMRD) open data site [8]. Water bodies data, including main rivers and lakes, were sourced from the East Africa Water Bodies dataset on RCMRD [9]. To ensure consistency of the spatial resolution with population data, all other raster data were resampled to 100-meter resolution.

**Appendix S2. Calculating travel times.**

To calculate the shortest travel times, we utilized AccessMod (5.8.3) to create a cost-friction surface by rasterizing the road network and water bodies and merging them with land cover data. As outlined in Supplementary Figure S2, we assigned specific speeds and modes of transportation to each road type and land cover category based on previous studies [4, 6, 10]. Each grid cell in the cost-friction surface corresponds to a particular land cover or road type, with travel speeds and modes determined accordingly. In cases where the road network and land cover overlap, the speeds and transport modes from the road network were selected for the corresponding grid cells. To compute the travel time cost for each grid cell, we divided the cell resolution (100 meters) by the assigned travel speed. This computation process returned the time required to cross each cell in either the north/south or east/west direction on the cost-friction surface, independent of the specific path taken [11].

To further refine our analysis, we incorporated slope data derived from DEM to account for terrain effects on walking and bicycling travel times. Walking speeds were adjusted according to the formula established by Tobler [12]; while bicycling speeds were modified through power adjustment methods [13]. It is noteworthy that the motorized mode is applicable to local transportation such as Boda Boda and Matatus [14, 15], where no modifications are made to their traveling speed. Additionally, we treated water bodies as barriers to movement, except in locations where bridges were present.

Finally, by integrating the cost-friction surface with the locations of vaccination centers, the cost path distance algorithm computed the cumulative travel time from each grid cell to the nearest vaccination site [11]. It produced a travel time surface, where each grid cell represents the shortest travel time for individuals to reach vaccination centers from that location. All maps and spatial visualizations in this study were created using ArcGIS Pro 3.4.0.

## Appendix S3. Justification for covariate selection

Numerous factors influence COVID-19 vaccination uptake. Younger individuals often exhibit lower willingness to be vaccinated because they perceive themselves at lower risk of severe disease, although some studies report higher acceptance among youth in certain settings [16–18]. Women tend to display lower or delayed uptake, frequently associated with concerns about vaccine safety [19, 20]. Parents or guardians may hesitate owing to logistical challenges in scheduling and attending vaccination appointments while balancing childcare and household responsibilities [21, 22]. Information access may shape vaccine attitudes [23]. In Kenya, recent internet use correlates with increased hesitancy, likely driven by misinformation on social media [24, 25]. Employment-related vaccination mandates also promote uptake to safeguard jobs [26], while having regular healthcare providers enhances likelihood of vaccination through exposure to credible medical advice and resources [22]. Higher trust in government is likewise associated with greater vaccine acceptance [27]. Educational attainment is another key determinant of vaccination: a recent global analysis found that tertiary education showing the strongest positive association with acceptance and uptake [28]. Wealth also plays a substantial role: a systematic review and meta-analysis in LMICs found that individuals in the poorest wealth quintile were 27% less likely to be fully vaccinated than those in the richest [29], motivating inclusion of a household wealth index to capture socioeconomic gradients in COVID-19 vaccine uptake. Finally, we adjusted for urban residence because urban–rural differences in service availability, infrastructure, and health-seeking behavior are well documented and may confound the relationship between geographic access and vaccination outcomes.

## Appendix S4. Results from Sensitivity analyses

*Characteristics of excluded respondents*

We first compared respondents excluded from the restricted analytic sample (n = 135) with those retained (n = 1,191) to characterize the subgroup removed by the exclusion criteria (Supplementary Table S1). Excluded respondents were younger (mean age 31 vs 33 years; $p = 0.024$), more likely to have primary or below education (24% vs 15%), and less likely to have post-secondary education (25% vs 48%; $p < 0.001$). They were from poorer households (mean wealth index −0.24 vs 0.03; $p = 0.002$), were more often uninsured (56% vs 38%; $p < 0.001$), and more frequently reported no known COVID-19 cases in their social networks (69% vs 47%; $p < 0.001$). There were no statistically significant differences between excluded and retained respondents in other characteristics. Overall, the exclusion criteria removed a subgroup characterized by younger age, lower educational attainment, lower household wealth, lower perceived infection risk, and lower household health insurance coverage. This pattern supports the interpretation that, for many of these respondents, reporting vaccines as "unavailable" despite living within 15 minutes of a site likely reflects barriers beyond geographic proximity (e.g. information gaps, perceived eligibility, or weaker engagement with the formal health system), and that the restricted sample more cleanly isolates the relationship between geographic access and the outcomes of interest.

Mixed-effects models

To assess the influence of unmeasured constituency-level heterogeneity, we re-fitted the four primary models using mixed-effects logistic regression with a random intercept for constituency, retaining the same fixed effects and covariates as in the main analyses. The overall pattern of associations was similar to that observed in the survey-weighted models (Supplementary Table S2). In the full analytic sample, travel time to the nearest vaccination site was not associated with either vaccine uptake or perceived vaccine availability. In the restricted sample, longer travel time remained strongly associated with both lower uptake and lower perceived availability, with clear evidence of non-linear effects (vaccine uptake: overall $p = 0.003$, nonlinearity $p = 0.008$; perceived availability: overall $p < 0.001$ for both overall association and nonlinearity). These findings suggest that the main results are not driven by unobserved constituency-level factors captured by a random intercept.

Grid-based travel time measures

We also evaluated sensitivity to the choice of travel time metric. Using the 100 m resolution travel time raster, we calculated for each constituency the mean and median

of the shortest travel time from all grid cells to the nearest vaccination site. These grid-based mean and median travel times were then assigned to respondents and substituted for the centroid-based measure in otherwise identical survey-weighted models (Supplementary Table S3).

For vaccine uptake, the overall association between travel time and uptake was not statistically significant in any specification. Tests for nonlinearity were generally non-significant as well; only in the full-sample model was there weak evidence of nonlinearity ($p = 0.078$), but in that case the overall association between travel time and uptake remained non-significant. For perceived vaccine availability, associations with travel time were likewise not statistically significant in the full sample. In the restricted sample, however, longer travel time remained strongly and nonlinearly associated with lower perceived availability, regardless of whether mean or median grid-based travel time was used (overall $p < 0.001$; nonlinearity $p \leq 0.003$). These results indicate that the association between travel time and perceived availability is robust to alternative definitions of the travel time exposure, whereas associations with vaccine uptake are more sensitive to how travel time is operationalized.

**Appendix S5. Supplementary Figures and Tables.**

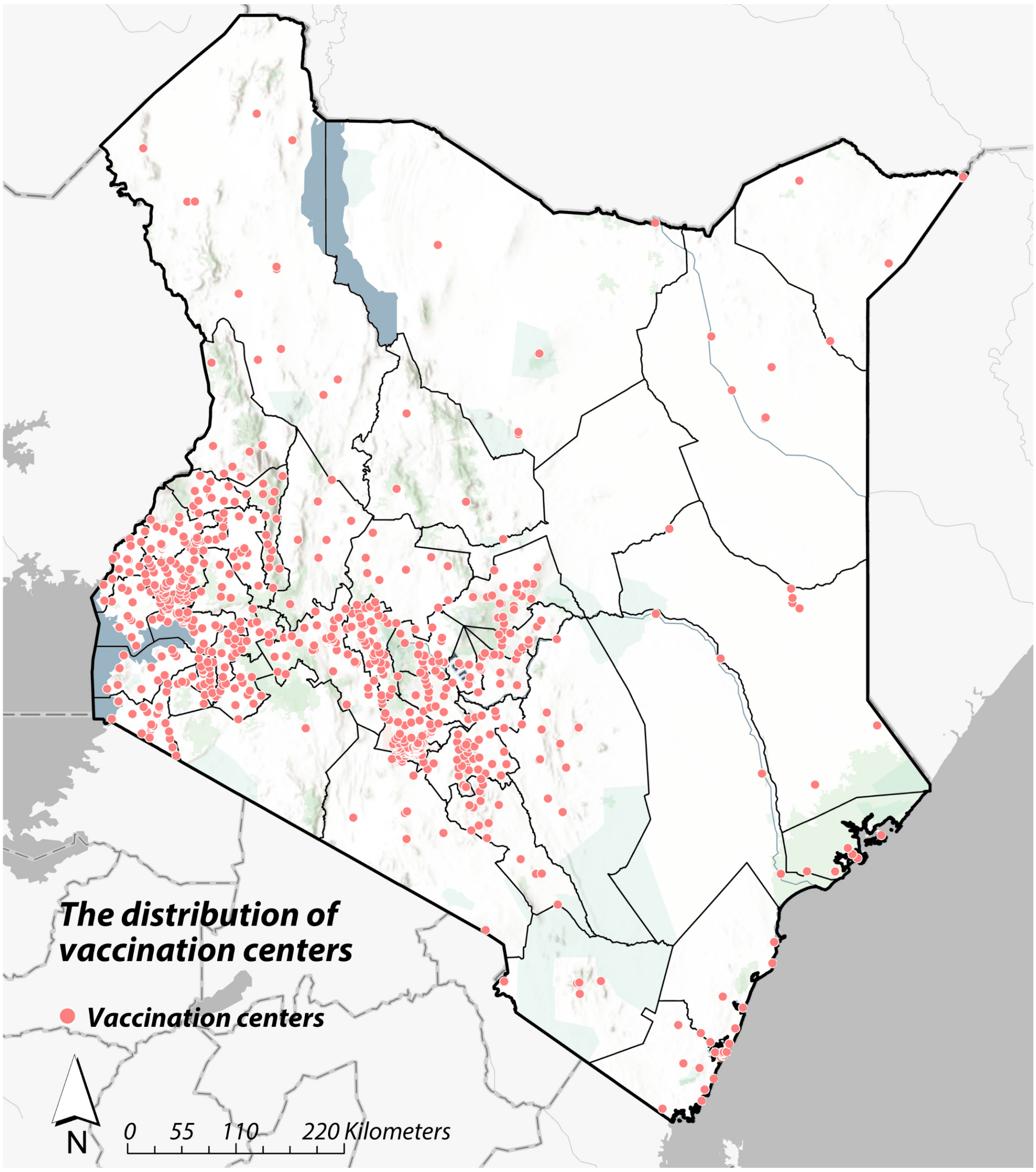


**Figure S1**. Distribution of COVID-19 vaccination sites across Kenya.

| Classification | Speed (km/h) | Transport mode |
|---|---|---|
| **Land cover** | | |
| Water | 0 | Walking |
| Trees | 2.5 | Walking |
| Flooded vegetation | 0 | Walking |
| Crops | 4 | Walking |
| Built area | 5 | Walking |
| Bare ground | 5 | Walking |
| Snow/Ice | 2.5 | Walking |
| Rangeland | 4.5 | Walking |
| **Road type** | | |
| Rural road | 5 | Walking |
| Country road | 10 | Bicycling |
| Secondary road | 30 | Motorized |
| Primary road | 50 | Motorized |

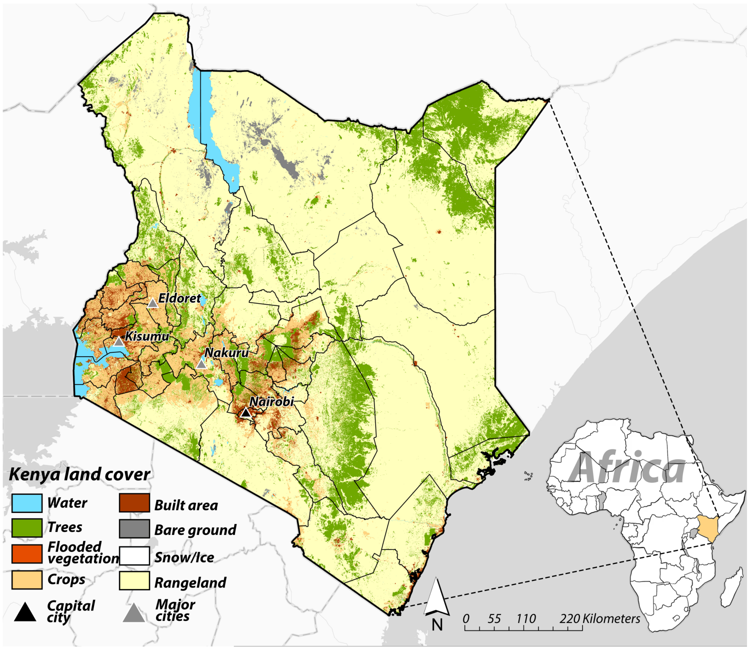


**Figure S2**. Hypothesized modes of transportation and corresponding speeds for residents traveling across different land covers or road types and land cover distribution across Kenya.

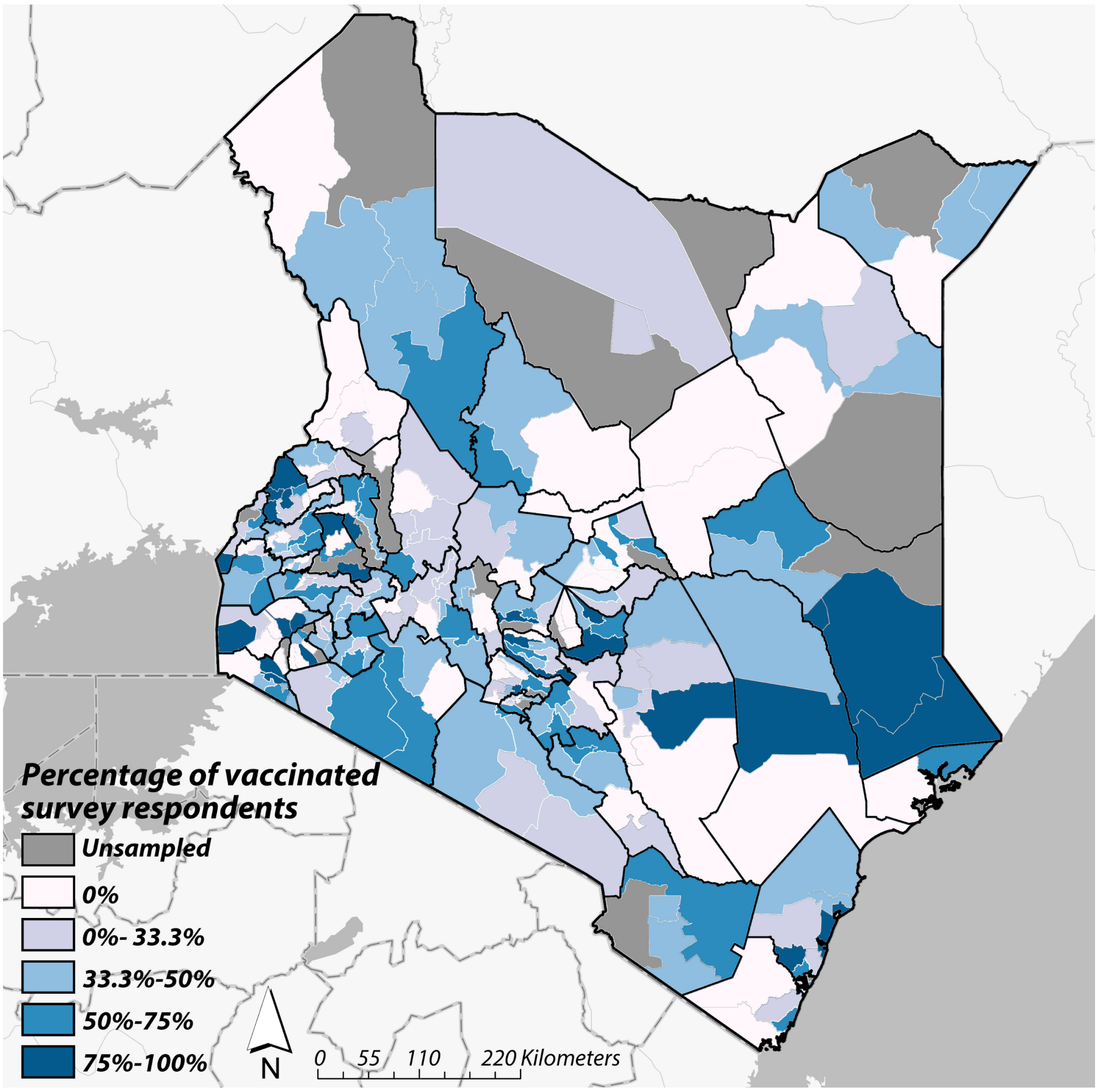


**Figure S3**. Percentage of survey respondents who have been vaccinated across Kenya.

**Supplementary Table S1**. Characteristics of Respondents Excluded From and Retained in the Restricted Analytic Sample

| Characteristic | Excluded<br>N = 135[1] | Retained<br>N = 1,191[1] | p-value[2] |
|---|---|---|---|
| Age | 31 (9) | 33 (11) | 0.024 |
| Perceived Infection Risk | | | <0.001 |
| No | 93 (69%) | 558 (47%) | |
| 1-2 | 20 (15%) | 257 (22%) | |
| 3+ | 22 (16%) | 376 (32%) | |
| Job Loss Experience | | | 0.13 |
| No | 79 (59%) | 776 (65%) | |
| Yes | 56 (41%) | 415 (35%) | |
| Children in the Household | | | 0.1 |
| No | 48 (36%) | 329 (28%) | |
| 1-2 | 48 (36%) | 523 (44%) | |
| 3+ | 39 (29%) | 339 (28%) | |
| Internet Connectivity | | | 0.5 |
| Without Household Internet | 116 (86%) | 997 (84%) | |
| With Household Internet | 19 (14%) | 194 (16%) | |
| Educational Attainment | | | <0.001 |
| Primary or Below | 32 (24%) | 173 (15%) | |
| Secondary | 69 (51%) | 450 (38%) | |
| Post-secondary | 34 (25%) | 568 (48%) | |
| Trust in Government | | | 0.9 |
| Strongly Distrust | 24 (18%) | 174 (15%) | |
| Somewhat Distrust | 17 (13%) | 138 (12%) | |
| Neither Trust nor Distrust | 7 (5.2%) | 67 (5.6%) | |
| Somewhat Trust | 45 (33%) | 418 (35%) | |
| Strongly Trust | 42 (31%) | 394 (33%) | |
| Insurance Coverage | | | <0.001 |
| Not Insured | 75 (56%) | 449 (38%) | |
| Insured | 60 (44%) | 742 (62%) | |
| Residence | | | 0.3 |
| Rural Residence | 71 (53%) | 571 (48%) | |
| Urban Residence | 64 (47%) | 620 (52%) | |
| Wealth | -0.24 (1.03) | 0.03 (0.99) | 0.002 |
| Gender | | | 0.2 |
| Female | 76 (56%) | 740 (62%) | |
| Male | 59 (44%) | 451 (38%) | |

[1] Mean (SD); n (%) [2] Wilcoxon rank sum test; Pearson's Chi-squared test

**Supplementary Table S2**. Associations Between Travel Time, Perceived Vaccine Availability, and Vaccine Uptake in Mixed-Effects Logistic Regression Models

| Sample | Outcome | Test Type | Df | Chisq | p-value |
|---|---|---|---|---|---|
| Full sample | Perceived vaccine availability | Nonlinearity | 2 | 5.0501 | 0.08005 |
| Full sample | Perceived vaccine availability | Overall association | 3 | 5.0542 | 0.1679 |
| Full sample | Vaccine uptake | Nonlinearity | 2 | 4.1251 | 0.1271 |
| Full sample | Vaccine uptake | Overall association | 3 | 4.5608 | 0.2069 |
| Restricted sample | Perceived vaccine availability | Nonlinearity | 2 | 70.976 | <0.001 |
| Restricted sample | Perceived vaccine availability | Overall association | 3 | 89.178 | <0.001 |
| Restricted sample | Vaccine uptake | Nonlinearity | 2 | 9.7599 | 0.008 |
| Restricted sample | Vaccine uptake | Overall association | 3 | 13.931 | 0.003 |

**Supplementary Table S3**. Associations Between Travel Time and Vaccine Outcomes Using Grid-Based Mean and Median Travel Time Measures

| Sample | Outcome | Travel Time | Test Type | Statistic | Value | df | p-value |
|---|---|---|---|---|---|---|---|
| Full sample | Perceived vaccine availability | Gridded-level Mean | Overall association | F | 0.5866 | 3,235 | 0.62432 |
| Full sample | Perceived vaccine availability | Gridded-level Mean | Nonlinearity | 2logLR | 0.6393 | — | 0.72487 |
| Full sample | Perceived vaccine availability | Gridded-level Median | Overall association | F | 0.8596 | 3,235 | 0.46273 |
| Full sample | Perceived vaccine availability | Gridded-level Median | Nonlinearity | 2logLR | 1.3318 | — | 0.51289 |
| Full sample | Vaccine uptake | Gridded-level Mean | Overall association | F | 1.613 | 3,235 | 0.18704 |
| Full sample | Vaccine uptake | Gridded-level Mean | Nonlinearity | 2logLR | 5.1633 | — | 0.078434 |
| Full sample | Vaccine uptake | Gridded-level Median | Overall association | F | 1.6256 | 3,235 | 0.18412 |
| Full sample | Vaccine uptake | Gridded-level Median | Nonlinearity | 2logLR | 5.1801 | — | 0.077765 |
| Restricted sample | Perceived vaccine availability | Gridded-level Mean | Overall association | F | 5.8029 | 3,233 | <0.001 |
| Restricted sample | Perceived vaccine availability | Gridded-level Mean | Nonlinearity | 2logLR | 13.1362 | — | <0.003 |
| Restricted sample | Perceived vaccine availability | Gridded-level Median | Overall association | F | 6.2471 | 3,233 | <0.001 |
| Restricted sample | Perceived vaccine availability | Gridded-level Median | Nonlinearity | 2logLR | 14.5942 | — | <0.002 |
| Restricted sample | Vaccine uptake | Gridded-level Mean | Overall association | F | 1.269 | 3,233 | 0.28573 |
| Restricted sample | Vaccine uptake | Gridded-level Mean | Nonlinearity | 2logLR | 4.0614 | — | 0.1342 |

| | | | | | | | |
|---|---|---|---|---|---|---|---|
| Restricted sample | Vaccine uptake | Gridded-level Median | Overall association | F | 1.1377 | 3,233 | 0.33458 |
| Restricted sample | Vaccine uptake | Gridded-level Median | Nonlinearity | 2logLR | 3.6535 | — | 0.16397 |

## References


[1] Google. Geocoding API of google maps platform, https://developers.google.com/maps/documentation/geocoding/ (2021, accessed 23 May 2025).

[2] WorldPop. Global 100m Population. Epub ahead of print 2018. DOI: 10.5258/SOTON/WP00645.

[3] OCHA Regional Office for Southern and Eastern Africa (ROSEA). Kenya - Subnational Administrative Boundaries, https://data.humdata.org/dataset/cod-ab-ken (2023, accessed 21 September 2025).

[4] Joseph NK, Macharia PM, Ouma PO, et al. Spatial access inequities and childhood immunisation uptake in Kenya. *BMC Public Health* 2020; 20: 1407.

[5] Macharia PM, Odera PA, Snow RW, et al. Spatial models for the rational allocation of routinely distributed bed nets to public health facilities in Western Kenya. *Malar J* 2017; 16: 367.

[6] Moturi AK, Suiyanka L, Mumo E, et al. Geographic accessibility to public and private health facilities in Kenya in 2021: An updated geocoded inventory and spatial analysis. *Front Public Health*; 10. Epub ahead of print 3 November 2022. DOI: 10.3389/fpubh.2022.1002975.

[7] Zanaga D, Van De Kerchove R, Daems D, et al. ESA WorldCover 10 m 2021 v200. Epub ahead of print 28 October 2022. DOI: 10.5281/zenodo.7254221.

[8] Regional Center for Mapping of Resources for Development (RCMRD). Kenya SRTM DEM 30meters, https://opendata.rcmrd.org/datasets/rcmrd::kenya-srtm-dem-30meters/ (2018, accessed 21 September 2025).

[9] Regional Center for Mapping of Resources for Development (RCMRD). East Africa Water bodies, https://opendata.rcmrd.org/datasets/rcmrd::east-africa-water-bodies/ (2023, accessed 21 September 2025).

[10] Macharia PM, Mumo E, Okiro EA. Modelling geographical accessibility to urban centres in Kenya in 2019. *PLOS ONE* 2021; 16: e0251624.

[11] AccessMod. Calculation details - Accessibility analysis (Appendix 3). In: *AccessMod user manual*, https://accessmod.atlassian.net/wiki/spaces/EN/pages/4325817/Appendix+3+-+Calculation+details+-+Accessibility+analysis (2024, accessed 23 May 2025).

[12] Tobler WR. *Three Presentations on Geographical Analysis and Modeling: Non-isotropic Geographic Modeling; Speculations on the Geometry of Geography; and Global Spatial Analysis*. NCGIA, University of California, 1993.

[13] Austin C. Bike Calculator, http://bikecalculator.com/ (2023, accessed 23 May 2025).

[14] Mkutu K, Mkutu TR. Public health problems associated with "boda boda" motorcycle taxis in Kenya: The sting of inequality. *Aggress Violent Behav* 2019; 47: 245–252.

[15] Mutongi K. *Matatu: A History of Popular Transportation in Nairobi*. University of Chicago Press. Epub ahead of print 2017. DOI: 10.7208/chicago/9780226471426.001.0001.

[16] Lazarus JV, Wyka K, Rauh L, et al. Hesitant or Not? The Association of Age, Gender, and Education with Potential Acceptance of a COVID-19 Vaccine: A Country-level Analysis. *J Health Commun* 2020; 25: 799–807.

[17] Solís Arce JS, Warren SS, Meriggi NF, et al. COVID-19 vaccine acceptance and hesitancy in low- and middle-income countries. *Nat Med* 2021; 27: 1385–1394.

[18] Troiano G, Nardi A. Vaccine hesitancy in the era of COVID-19. *Public Health* 2021; 194: 245–251.

[19] Ba MF, Faye A, Kane B, et al. Factors associated with COVID-19 vaccine hesitancy in Senegal: A mixed study. *Hum Vaccines Immunother* 2022; 18: 2060020.

[20] Paudel YR, Du C, MacDonald SE. COVID-19 vaccine coverage among immigrants and refugees in Alberta: A population-based cross-sectional study. *J Glob Health* 2022; 12: 05053.

[21] Barry V. Patterns in COVID-19 Vaccination Coverage, by Social Vulnerability and Urbanicity — United States, December 14, 2020–May 1, 2021. *MMWR Morb Mortal Wkly Rep*; 70. Epub ahead of print 2021. DOI: 10.15585/mmwr.mm7022e1.

[22] Guay M, Maquiling A, Chen R, et al. Measuring inequalities in COVID-19

vaccination uptake and intent: results from the Canadian Community Health Survey 2021. *BMC Public Health* 2022; 22: 1708.

[23] Chai R, Yang J, Su R, et al. Low uptake of COVID-19 booster doses among elderly cancer patients in China: A multicentre cross-sectional study. *J Glob Health* 2024; 14: 05010.

[24] Amodan BO, Okumu PT, Kamulegeya J, et al. Knowledge, attitudes and barriers to uptake of COVID-19 vaccine in Uganda, February 2021. *BMJ Glob Health*; 10. Epub ahead of print 26 March 2025. DOI: 10.1136/bmjgh-2024-016959.

[25] Cuan-Baltazar JY, Muñoz-Perez MJ, Robledo-Vega C, et al. Misinformation of COVID-19 on the Internet: Infodemiology Study. *JMIR Public Health Surveill* 2020; 6: e18444.

[26] Maughan-Brown B, Eyal KC, Njozela L, et al. Predictors of COVID-19 vaccine uptake among adults in South Africa: multimethod evidence from a population-based longitudinal study. *BMJ Glob Health*; 8. Epub ahead of print 4 August 2023. DOI: 10.1136/bmjgh-2023-012433.

[27] Moola S, Gudi N, Nambiar D, et al. A rapid review of evidence on the determinants of and strategies for COVID-19 vaccine acceptance in low- and middle-income countries. *J Glob Health* 2021; 11: 05027.

[28] Lupu D, Tiganasu R. Does education influence COVID-19 vaccination? A global view. *Heliyon*; 10. Epub ahead of print 15 February 2024. DOI: 10.1016/j.heliyon.2024.e24709.

[29] Ali HA, Hartner A-M, Echeverria-Londono S, et al. Vaccine equity in low and middle income countries: a systematic review and meta-analysis. *Int J Equity Health* 2022; 21: 82.
